# Exploring the potential of a school brushing program using a connected brush in underserved areas: a feasibility cluster randomised trial

**DOI:** 10.1186/s12903-025-05573-7

**Published:** 2025-02-11

**Authors:** George Kitsaras, Nicola Boothman, Juliana Gomez, Michaela Goodwin, Momina Muzammil, Tanya Walsh

**Affiliations:** 1 Division of Dentistry, University of Manchester, Manchester, UK; 2 Colgate-Palmolive Company, Dental Health Unit, Williams House, Manchester Science Park, Manchester, M15 6SE UK

**Keywords:** Oral health, Children, Dental caries, Oral hygiene, Technology, Text messages, Connected technology

## Abstract

**Background:**

The main aim of this cluster randomised controlled trial is to explore the feasibility of a school-based brushing program utilising a smart, connected brush in children.

**Methods:**

Students aged 8 to 11 years in 6 primary schools across Manchester, UK were approached for this study. All children received a smart, connected toothbrush which captured information on coverage, duration and frequency of brushing. Teachers had access to class-level digital dashboards summarising brushing habits to set challenges for the students. Additionally, children in schools randomised to the toothbrush plus text messaging trial arm received text message support (delivered to their parents' mobile phones). Outcomes included: (a) consent rate, (b) recruitment rate recorded as the number of participants who completed at least one of the study surveys post-consents, and (c) acceptability of the interventions on schools and parents through a mixed methods approach.

**Results:**

Average age of parents was 39.8 years (SD5.94) while the average age of children was 9.7 years (SD1.56). Most parents and children came from households in the most deprived decile based on their Index of Multiple Deprivation (IMD) scores with 77 households (52.4%) scoring 1 (most deprived). Out of 753 eligible participants across all 6 schools, 409 were consented (54.3% consent rate) with 148 participants completing baseline measurements (36.8%). During the study, there was only 1 drop out. In terms of acceptability, parents found the study very enjoyable (average score of 8.9/10), and found the brush and app easy to use (average score of 8.7/10). Those parents who additionally received the text messages found them easy to understand and useful (average score of 8.9/10). In total, 91.6% of parents would recommend the brush and the intervention to family and friends. Three key facilitators ((a) enhancing children’s self-reliance, (b) increased engagement through the use of visual cues and (c) increased motivation (due to gamification) potentially supported children’s engagement with the brush and app.

**Conclusions:**

Low dropout rates and very positive feedback highlight the potential of this intervention. Future studies should consider how to quantify changes in children’s oral health, address loss of questionnaire completion and harness the opportunities this study provided.

**Trial registration:**

The study was registered at ISRCTN registration number ISRCTN77803149 on the 28th December 2023.

**Supplementary Information:**

The online version contains supplementary material available at 10.1186/s12903-025-05573-7.

## Background

Dental caries in children remain an important public health issue [1]. Dental caries can have adverse, lifelong implications [1]. Burden of disease associated with dental caries is one of the highest around the world contributing to billions of healthcare costs with detrimental personal and societal effects [2] In England, overall prevalence of dental caries is reducing however, pockets of high disease in underserved areas remain [3]. For example, many local authorities across the Northwest of England such as Greater Manchester (GM) have a higher prevalence of dental disease for 5-year-olds than the national average [3]. Dental caries in children can result in short- and long-term negative implications including pain, chewing difficulties [4], sleeping difficulties [5], changes in behaviour (e.g., irritability), adverse psychological development (such as low self-esteem), and loss of school days with an associated impact on school performance [6–8]. If left untreated, dental caries can progress and lead to extractions under general anaesthetic (GA) in hospital [5] with the NHS spending £81 m on preventable tooth extractions in young children per annum [1, 2, 4].

Dental caries is a preventable, multifactorial disease through adequate and frequent oral hygiene practices including brushing teeth twice a day, at bedtime and another occurrence through the day for example, in the morning, reducing frequency and amount of sugar consumed during the day, flossing, and using mouthwash as well as registering and attending dental professionals regularly for dental checks [1]. Individual, group and community-based interventions have been effective in preventing and/or slowing progression of dental disease in children [9, 10]. School brushing programmes have been successful in lowering the incidence of dental caries, however, there are significant barriers to implementation [11]. School brushing programs require additional time in the curriculum, cross infection control and hygiene measures (especially since the COVID-19 pandemic) and pose significant logistical challenges leading to a steady decline in the number of schools offering daily toothbrushing [12].

Outside of school brushing programmes, there are multiple ways to help parents establish good routines around oral hygiene and diet, many of which are cost-effective and utilise technological solutions. Helping parents to establish good oral health routines and dietary habits with their children is crucial. Family life revolves around routines from morning to bedtime. Family routines are crucial for child development and wellbeing as a multitude of beneficial behaviours and activities take place during family routines [13, 14]. Connected toothbrushes are part of emerging technologies that offer potential in supporting optimal oral health routines for people of different ages [15]. These devices can help provide real-time information to users, help them adjust their brushing technique to achieve better results, offer gamification elements to keep users, especially children, more engaged in the activity and offer challenges to reach greater oral hygiene goals, for example, brushing for longer and closer to 2-min each time, covering more areas around the mouth and brushing twice a day every day.

Alongside connected technologies, there are other technology-focused tools to help families and children to establish good routines around oral hygiene and diet. Text message-based solutions can also support parents and children in establishing optimal oral hygiene routines [16]. Text messages can be delivered to all mobile phone devices including non-smartphones. In the USA, Text4Baby [16] is an example of text messaging programmes offering succinct, targeted, and tailored advice to families with young children. In the UK, text message-based interventions have been successful in helping some parents establish better bedtime routines including oral health routines at bedtime [17].

To date, school-based oral health intervention combining the potential of using connected brushes with text message support for establishing better oral hygiene routines remains limited. Some studies have explored the use of electric brushes in school [18] however, these studies did not incorporate smart, connected brushes with a robust behavioural intervention to explore changes in oral health behaviours in children. It is important to explore if we can replace the need for active toothbrushing in schools while maintaining an element of overall school involvement in supporting children’s oral health. Further research should explore how connected brushes can support teachers in facilitating oral health conversations with students and if text messages can offer an additional layer of dynamic support at home.

Dental caries remains an enduring problem for children, and alongside the limitations surrounding traditional school brushing programs, there is a clear opportunity for technological solutions to be considered in tackling dental disease, instilling better oral health behaviours for children, and helping families.

## Aim & objectives

The main aim of this study was to explore the feasibility of a school-based brushing program utilising a smart, connected brush in children, with and without text messaging. This aim was achieved through the following objectives: (a) exploring consent rate of students in this programme, (b) understanding recruitment and retention rate, (c) assessing recruitment pathways for future studies and (d) exploring acceptability of the intervention for parents/students and teachers involved in the project.

## Methods

### Trial design

This was a feasibility cluster randomised controlled trial. All study activities were conducted between March and August 2023. The CONSORT 2010 extension for reporting pilot and feasibility randomised control trials has been followed throughout. Ethical approval was secured by the University of Manchester’s Proportionate UREC (Ref: 2022–15413-26,189). Appendix 1 provides a copy of the checklist.

### Participants & sample size

Students aged 8 to 11 years in 6 primary schools (in England, all children aged 5 to 11 must attend primary school education) across Greater Manchester were recruited for this study. Inclusion criteria were: (a) children enrolled and attending primary schools in Greater Manchester aged 8 to 11 years, (b) availability of WIFI in their home and a smart device that is compatible with the toothbrush and app (i.e., access to Apple iPhone (newer than 8) or iPad (newer than 5th generation) or Android users (running Android 6.0 and up) with access to an Internet connection) and (c) no prior use of the brush that is distributed during the study. Schools were identified due to their dental needs from Manchester City Council’s (MCC) Oral Health Improvement Team (OHIT). MCC OHIT identified areas with the highest prevalence of dental disease in children and they made first contact with potential schools to discuss the proposed study and explore their willingness to participate. Initial contact from OHIT was followed up with in person visits to each school by the research team to explain the study and answer any questions. Once schools confirmed their participation, all eligible students were informed about the study, provided with participant information sheets to take home so their parents could review and offered a few days to decide if they would like to take part in the study. Consent was obtained electronically using Qualtrics with the option for paper-based consent for those parents who did not want to consent online, for children, assent was obtained. In some schools, a morning information session was hosted so research staff could speak directly to parents about the study to answer any of their questions. Participants who completed at least one of the study surveys, received an online voucher (worth £50) as a thank you for their time, all participants were able to keep their connected brushes at the end of the study. All study materials were translated into the six most commonly spoken languages in the target areas (Urdu, Polish, Bengali, Pashto, Dari, Arabic).

At the end of the intervention period, participants who consented to be contacted for future research were approached over email for in-depth, online feedback interviews.

### Sample size

As the study was a feasibility trial, a formal power calculation based on hypothesis testing and detecting evidence for effectiveness was not appropriate. Given school sizes in targeted areas as identified based on their dental need from MCC’s OHIT, a sample of six school comprising between 400 and 600 eligible pupils was anticipated. This sample size was pragmatically chosen to allow us to). This was a pragmatically chosen sample, reflective in characteristics of the target population for a full trial, that would be sufficient to allow the team to identify challenges principally concerning recruitment and feasibility of delivery of the intervention, and data collection.

Qualitative, feedback interviews were completed with 20 parents across the six schools and data saturation was achieved. Participants were stratified according to each participating school, in an effort to reach equal distribution of participants from each school.

### Interventions & randomisation

All children (regardless of consent) received a hum by Colgate smart, connected toothbrush to use each day at home (morning and bedtime). The brush captures information on coverage, duration and frequency of brushing, communicating this data to children while they brush via an accompanying app. Apart from the brush, teachers had access to a brief, class-level dashboard where they saw how their class was doing (in comparison with other classes) and set challenges for the children to help them achieve better goals (or maintain good practices if they did well). Additionally, children in schools randomised to the toothbrush plus text messaging trial arm received text message support (delivered to their parents' mobile phones). Text messages contained messages directed at parents and children to emphasize key oral health promotion messages and to motivate them to continue using the brush. The COM-B model of behaviour change [19] was followed in developing aspects of this intervention. The provision of toothbrushes aimed to create opportunity through environmental restructuring and resources, information on oral health provided via the app and associated study documents created capacity, especially psychological capacity, while in-class challenges and social comparisons aimed to enhance motivation to undertake and follow the target behaviour (e.g., oral hygiene practices) for longer.

All intervention elements were delivered for 3 months (follow-up period). Brushes were used at home directly by children with parental supervision. Teachers received access to the weekly dashboard on Monday of each week. In the text message trial arm, parents received messages in a tapered approach with more text messages at the start of the study and less towards the end. In total, each parent/ caregiver received nine text messages over the course of the study. Appendix 2 presents the logic model of the intervention.

### Randomisation

Allocation to treatment arms was at the school level (simple randomisation [flipping a coin]) with participating schools receiving either the connected brush and text messages or receiving only the connected brush.

### Outcomes

Primary outcomes focused on feasibility of using a smart, electric brush by children, assessed using: (a) consent rate recorded as the number of participants consented at 2 weeks following the initial approach by the study team, (b) recruitment rate defined as the number of participants who completed at least one of the study surveys post-consent (post-baseline), (c) recruitment pathways for future studies recorded as the number of new sites (first time involved in research studies) taking part in the study and (d) acceptability of the interventions on schools (teachers) and parents recorded at follow-up through surveys and qualitative feedback interviews.

Additional, secondary outcomes included: (a) Knowledge, Attitudes and Practices regarding oral health measures by a brief questionnaire at baseline and end of follow-up and (b) Oral hygiene practices measured by a brief oral hygiene survey at baseline, 1-week, 1-month, 2-month and 3-month follow-up. These outcomes ((a) and (b)) have been summarised in a separate publication which also included information captured by the smart, connected brush on coverage, frequency, and duration. All quantitative data collection took place electronically using Qualtrics.

Qualitative interviews followed a semi-structured interview guide. Questions were developed to explore key barriers and facilitators regarding the implementation of the intervention in school following the Theoretical Domains Framework (TDF) [20]. Qualitative, feedback interviews were conducted online on Zoom. Participants were given an option to take the interview in English (19 participants) or Urdu (1 participant). The transcriptions were done using a transcription company, except the interview in Urdu (Participant 10), which was translated and transcribed by the interviewer who is a native speaker (MM).

### Analyses

Consent and recruitment rates were analysed descriptively. Loss to follow up and data collected on how often participants sync the toothbrush to the app were reported. Quantitative analysis was carried out for the scale-based responses to the Knowledge, Attitudes and Practices (KAPs) questionnaire and brief oral hygiene survey. Feedback surveys were also analysed descriptively to summarise participants’ views on the intervention and gather important feedback for future progression to a definite trial.

A deductive thematic analysis was carried out to identify the main facilitators and barriers to using the connected brush and associated app. Two coders (GK and MM) read a set of interviews and developed an initial coding key. All transcripts were read and coded according to the initial coding key with adjustments to the codes throughout the process. All codes across all interviews were collated and searched for repetitions and patterns. Codes that fit together (like happy, positive, fun etc.) were merged together in a theme. Themes were reviewed and defined before completing the analysis.

## Results

### Sample characteristics

**Fig. 1:**
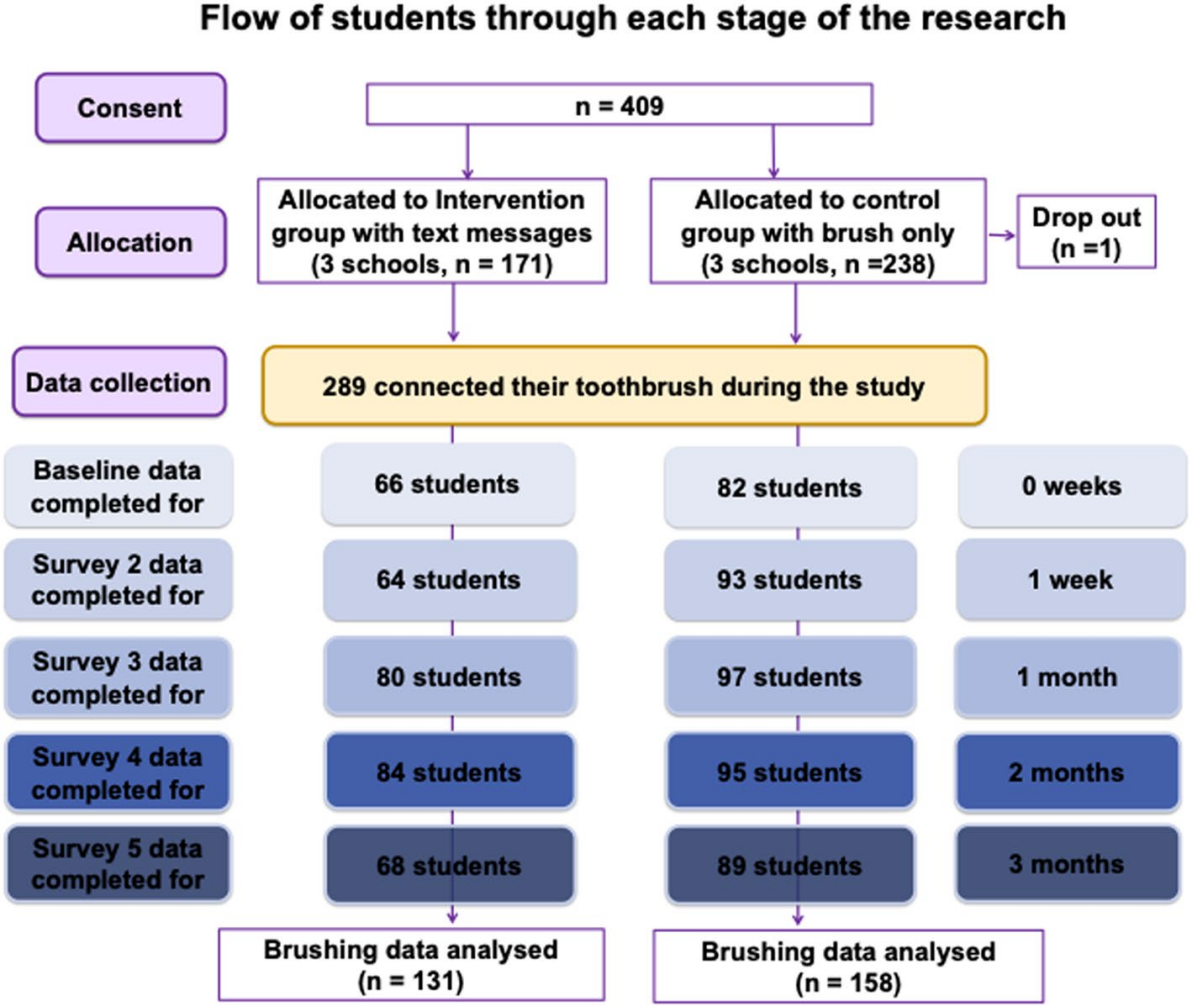
Participants’ flow

Six schools across underserved areas of Manchester were identified and approached to participate in the study. All consented and were randomised to the brush and app trial arm or brush, app and text message arm. Of 753 eligible children, 409 (54.3%) participants consented to take part in the study. Out of those, 171 (41.80% of total participants) in three schools were allocated to the intervention group with the text messages. Out of 409 consented participants, 148 completed baseline measurements, 157 1-week follow-up measurements, 177 1-month follow-up measurements, 179 2-month follow-up measurements, 157 3-month follow-up measurements. Figure 1 provides an overview of participants flow.

Among the 148 participants who completed baseline surveys, the mean age of parents was 39.8 (SD5.94) while the mean age of children was 9.7 years (SD 1.6). In total, 121 mothers and 27 fathers completed baseline surveys. The majority of parents was of white ethnic background (*N* = 70, 47.9% of total sample), followed by Asian/British-Asian ethnic groups (*N* = 38, 26% of total sample), 8.9% (or *N* = 13) participants were of Black/Black-British/Caribbean ethnic group while 25 participants (17.1%) were of multiple ethnic or other ethnic groups. The majority of parents had full English competency (speak, read, write) (*N* = 120, 81.6%) with 27 participants reporting English fluency in one or more domains (speak, read or write). Most parents were in full-time employment (*N* = 74, 50%), with some working part-time (*N* = 39, 26.4%), others being stay at home parents (*N* = 14, 9.5%), 10 parents were unemployed (6.8%), 4 were students (2.7%), 1 retired (< 1%) and 6 preferred not to provide their occupation (4.1%). Finally, most parents and children came from households in the most deprived decile based on their Index of Multiple Deprivation (IMD) [21] scores with 77 households (52.4%) identified as decile1 (most deprived). In total, 50 households (34%) came from the second most deprived decile (score of 2), 11 (7.5%) from the 3rd most deprived decline (score of 3) while no household in the study came from deciles over 5. The overall composition of our sample closely reflects that of key areas of higher disease prevalence across the city and the country as a whole. With dental disease prevalence reducing generally, pockets of disease remain in areas of higher deprivation which as the ones targeted by our research.

Out of 20 participants who completed the follow-up, feedback, qualitative interviews, 18 (90%) were female with 17 participants (85%) from the most deprived deciles (1 and 2). There was an equal split in terms of ethnicity with 50% of participants identifying as White.

### Feasibility outcomes

In terms of primary outcomes, out of 753 eligible participants across all 6 schools, 409 were consented (54.3% consent rate) with 148 participants completing baseline measurements (38.6%) as summarised in Fig. 1. Consent rates differed between participating schools with some recruiting as many as 67.8% of all eligible students while others consented around 42% of eligible students. Schools that hosted morning information sessions for parents did recruit more participants (mean 64.3 (SD4.95)) when compared to schools who did not host information sessions (mean47.0 (SD5.82)) (t(4) = 3.54, *p* = 0.023). The biggest reason for participants not completing their online baseline measurements was email addresses not working resulting in emails bouncing back (50 addresses not responding out of 409, 12% of consented participants). During the study, there was only 1 drop out due to inability to commit to study time commitments. All schools who participated in the survey were first-time research sites for similar research work with a very strong relationship developing between the research team, MCC OHIT and participating schools paving the way for future involvement in studies. Overall, an average of 164 participants provided data throughout the 5 follow-up points in the study reflecting 40.1% of all consented participants.

In terms of acceptability, parents found the study very enjoyable (average score of 8.9/10 in follow-up surveys), parents found the brush and app easy to use (average score of 8.7/10 in follow-up surveys). Parents of children in schools allocated to receive text messages also found them easy to understand and useful (average score of 8.9/10 in follow-up surveys) and finally, from the entire sample, most parents (*N* = 142, 91.6% of those completing feedback surveys) would recommend the brush and the intervention to family and friends.

Based on teacher’s feedback (provided through brief feedback surveys at the end of the study), they felt extremely valued and highly positive towards the intervention, specifically the use of the connected brush, app and the class performance tables. Teachers appreciated being part of this intervention and recognised the value of oral health for their students. Teachers reported some issues around connectivity in line with comments from parents. These issues seem to have caused some struggles for some students.

“…some students struggled to sync, it wasn’t that clear, we asked them to read the guidelines, but some still struggled…” (T3)

“…some students lost the information we provided, as it happens, so maybe circulating the information more often couId have been better…” (T4)

The overall time commitments required by teachers was not an issue for all of them and teachers also valued the deployment of the dashboard to help motivate classes to reach higher goals in their brushing frequency, coverage and duration. Also, some teachers found the use of a connected brush useful beyond the scope of the study by incorporating information from the brush into their lessons.

“(the) brush has helped with teaching students personal hygiene, I am big on personal hygiene and I’ve used the brush as a good prop to help students take care of their teeth” (T2)

### Barriers & facilitators to adopting the connected brush

Three key *facilitators* were uncovered during analyses of follow-up, feedback interviews with parents, namely: (a) enhancing children’s self-reliance, (b) increased engagement using visual cues and (c) increased motivation due to gamification.

- Developed adolescent children’s self-reliance in dental care

Most participants found the connected brush and app intervention helpful, primarily because it fostered self-reliance and responsibility in children. Children were taking responsibility of the device and their oral hygiene, and there was a shift in parental supervision to remote monitoring. Some reasons reported by the parents for the high engagement from children include the device’s novelty, gamification features (tracking brushing scores and immediate visual feedback through the app), and ease of use. Behavioural changes include children taking ownership of their routine, demonstrating responsibility by remembering essentials like chargers while traveling, and feeling more grown-up and independent.

*“So… she was really, really keen on making sure she was on track with it, and even when we’ve gone on holiday for a weekend, she wanted to make sure that she took her charger for the toothbrush and stuff. It gave her a really good sense of independence…. I think it made her feel a bit more grown up.”—Participant 14*

*“So yes, it was quite useful on both sides, to be honest, for both me and for her, a little bit more enthusiasm, you know, a bit of a gadget to play with, a little bit of enthusiasm for her to brush her teeth properly. So that was quite nice. I think for me it was interesting to see how she’d been doing, and to also encourage her to pay attention to what she does when she brushes her teeth, yes.” – Participant 19*

(b) Visual cues increase engagement and promote interactivity

Several participants mentioned the app provided clear, interactive and visually engaging cues that facilitated proper brushing, particularly providing real-time feedback. Parents said that visualising areas that the children were typically missing during routine toothbrushing or using visual cues to brush for a full two minutes both improved children’s toothbrushing. Visualizing their children’s toothbrushing was also helpful for parents. It improved their awareness as not all parents were aware of “how” to brush properly. It served as a useful tool for parental oversight as children grew independent while allowing parents to stay involved.

*“I think, me personally, I think that app and things, it gets the parent involved as well to make sure that they’re doing it, rather than being in school and, this is how you brush your teeth, type of thing. Because at least you can be both there watching the app and making sure that she doesn’t have any food or drink afterwards and things like that. I think, personally, something like that is a lot better than them just being taught in school.” Participant 15.*

It allowed parents to remotely monitor their child’s brushing, and helped build trust and accountability between the parent and child.

*“It broke my heart because she said that they had to take about eight teeth out…and she can't lie to me and say, yeah, I've done it. No you haven’t. So I thought that’s really good, and I'd recommend it to other people because I think it's brilliant.” (Participant 18).*

Visualizing the child’s brushing habits was also helpful for parents who felt “judged” by dental staff and felt their word was not taken seriously:

“…I think they think that she’s not cleaning her teeth, which was quite good because I could then show the dentist the app and say, look, she is, it's here, here’s the proof…” (Participant 18)

(c) Gamification and competition increase motivation in children

Gamification and goal-setting features which allowed children and parents to track progress, converted toothbrushing into a rewarding activity. Incorporating elements of play and achievement seemed to help children stay motivated. The sense of achievement kept children interested and motivated, and several children felt proud of it, reflected by them telling others about it, such as their dentist and cousins. Other features that kept children motivated were the novelty of the device, as most children had not used an electric toothbrush before. They were also motivated by competing against their siblings and other children at school.

*“Yeah. Well, the app does show, it shows points to show how much you’ve done. So, my children they keep tabs on each other. So X was saying to Y, ‘oh look, I have so many’. Y, I think when he missed it once or twice, so he had lesser points. So then between themselves, they kept feeling, like, ‘oh she has more, so I should quickly get mine up as well’ Participant 10.*

*“I am not 100 per cent sure, but I think when they were doing the three-month monitoring of it I feel like they did something at school as well. So that encouraged a lot of the other children because it became…they wanted to [set 15:27] the points, they wanted…it became more competitive, but in a nice way.” Participant 4*

Meanwhile, two key *barriers* ((a) unavailability of device to use the connected brush and (b) issues around safety and sustainability) were uncovered during analyses.

- Unavailability of device due to circumstance or preference

The availability of the phone or tablet to use the app was a major barrier. Most children were either using the app on their parent’s devices or their own tablets, which made regular app usage complicated particularly in the mornings, which tend to be busy for both parents and children. In addition, parents had concerns about children’s screen time, which tends to be a particular problem for adolescents.

*“No, because obviously it’s on my phone and I work sometimes and it’s more difficult. It wasn't connected to anything else, just my phone. So in the morning, am I rushed getting to work or am I not in work, it depends. So that time I was very…I was always getting late so I'm like, you know what, I'm just deleting it from my app, just do it on your own accord.” (Participant 13)*

“Do you know, I will say this one thing though, so the thing is with the app, we didn’t use it as much, ‘cause I’m trying to get my nine year old to not stay in the tablet, and when we take the tablet away from her, she’ll just [inaudible 00:19:42] phone, which I use, [to separate 00:19:45] my personal phone and the work phone, and she’ll just take my work phone and use that. So this is a bit of a challenge as well, I think from your point of view, if some parents don’t want to give their kids technology at that age, you know, ‘cause of the fear of social media and all that kind of stuff, and just sitting on a tablet mindlessly playing games, which is just brain dead.”

“We don’t really want to give her the phone, ‘cause she’ll use the phone for the app, and then she’ll just play a game with it later on.“ Participant 5

(b) Safety and sustainability concerns

There were other safety concerns that could have been addressed through detailed information leaflets and may have affected the reduced participation within the program. One participant mentioned other parents’ concerns about being “tracked” through the app.

*“But when this program was happening in my school, I remember people’s feelings, I remember it quite well. But mine were quite good. They were a bit scared that ‘oh, they’re going to control us through the device, they’re going to monitor us’. That was funny because I received phone calls. So children whose mothers couldn’t speak proper English, they called me, that you know, now they’re going to be monitored all the time. I told them no, they’ll just be able to see if they’re brushing their teeth. ‘No, that’s not right, they’ll be monitoring us’.” (Participant 10)*

Other concerns included the safety of electric toothbrushes in this age group and environmental concerns about the use of plastic, particularly due to the unavailability of reusable heads.

*“I guess I had asked our dentist what he thought about electric toothbrushes, and whether they were too abrasive for children or anything like that. So there must have therefore somewhere in the back of my mind being a bit of apprehension. But I think overall I was like, I thought it was a good thing.” (Participant 11)*

**“**…then came home with a plastic toothbrush, which we try and recycle. So it’s things like that that annoy me.” Participant 7

## Discussion

### Key outcomes: consent, response rates and retention

All feasibility outcomes were achieved at the end of the study with important lessons learnt for continuing this work in the future. We were able to recruit participants based on our original assumptions of 400 to 600 participants consented through 6 schools. Out of 753 eligible participants across all 6 schools, 409 were consented resulting in a 54.3% consent rate. Consent rates in research studies vary depending on the field and discipline. For example, a review of consent, recruitment, and retention of participants in randomised controlled trials found a median consent rate of 72% [22]. Generally, in children’s medical research, consent rates fluctuated from 42 to 94% [22]. A contemporary UK-based oral health intervention with secondary school pupils (adolescents) achieved 33% consent rate [23]. In comparison, the overall consent rate of this study is average and even higher than similar, school-based, oral health research undertaken in the UK. When comparing consent rates between schools who received additional support during recruitment (for example, by hosting a morning, information/feedback session for parents) average consent rates increased to 64.3% of all eligible students, closer a higher-than-average consent rate reported in published literature.

On average, 164 participants provided data during follow-up surveys representing 40.1% of all consented participants with 148 participants completing baseline measurements (36.2% of all consented participants). On average, 70–80% response rates are considered sufficient in questionnaire and survey studies [24]. Higher response rates can help mitigate non-response bias and desirability bias [25]. Non-response bias refers to the mistakes in estimating population characteristics and behaviours based on data in which specific populations, groups and individuals are under-represented due to them not responding to the survey in the first place [26]. Desirability bias refers to participants providing answers that are socially acceptable and expected versus the true nature of their experience and behaviours [27]. Desirability bias can be a common limitation of research studies especially when there are low response rates.

Different approaches can help increase response rates in research studies. Electronic data collection has been shown to be more effective in increasing participant questionnaire completion rates when compared to traditional, paper-based approaches. However, in our study, an unintended consequence of combining both paper-based and electronic consent processes led to email addresses not being clear to read in many paper-based consent forms leading to problems when trying to email participants for their baseline data completion.

For participants who were active in the study, there was only one active dropout throughout resulting in significant participant retention. This could reflect the low, overall time commitment and participant burden.

### Key outcomes: recruitment pathways

All sites which participated in our study were first-time research sites. They were all actively identified in partnership with the local OHIT. This approach led to the identification of schools who were most in need for the intervention based on their local level of disease prevalence. Also, working with OHIT meant we were able to align with any ongoing community-based oral health initiatives in the area to avoid interfering with any other programme. Community engagement is an essential part for successful community interventions. Local communities and local services often have a much more holistic and clear view of the health needs of a specific population or for a specific condition [28]. Incorporating community engagement in research studies and interventions can therefore act as level for sustained and effective change [28, 29]. Involving local communities and services in early stages of research can lead to their empowerment to become active collaborators and partners and to help achieve better, overall, and long-term outcomes [30]. There are now important foundations in place based on this initial, successful engagement with OHIT, as a community-based dental public health service, and inclusion of first-time research sites in our study.

### Key outcomes: acceptability of intervention

In terms of overall acceptability of the intervention provided and experience in taking part in the study, parents found the study very enjoyable (average score of 8.9/10 in follow-up surveys) and they also found the brush and app easy to use (average score of 8.7/10 in follow-up surveys). For those parents who received the text messages, they also found them easy to understand and useful (average score of 8.9/10 in follow-up surveys) and finally, most parents (*N* = 142, 91.6% of those completing feedback surveys) would recommend the brush and the intervention to family and friends. Positive participant experience is key for the success of any intervention as participants who find an intervention easy, attractive and practical will be more engaged resulting in better study outcomes [18]. The three key facilitators identified through follow-up, feedback interviews ((a) enhancing children’s self-reliance, (b) increased engagement using visual cues and (c) increased motivation due to gamification) showcase the potential of the intervention to result to initial positive engagement followed by future changes in key oral health behaviours. Oral hygiene behaviours are among one of the most common, repetitive health behaviours individuals need to engage with on a daily basis. One needs not only to know how to brush their teeth effectively but to also repeat the specific behaviours twice a day, multiple times a week and hundreds of times a month on repeat. Despite only taking a few minutes at a time, oral hygiene behaviours are not optimally achieved by all. Some studies have found around a quarter of children up to the age of 5 are not brushing their teeth twice a day as recommended [31] while others have found as many as 40% of children not brushing their teeth twice a day [32]. Therefore, interventions that aim to increase oral hygiene behaviours such as brushing in children need to be primarily easy and acceptable. Offering an engaging, interactive and relevant intervention can lead to higher rates of brushing behaviour for children which in turn can result in better oral health outcomes.

Meanwhile, the two key barriers identified ((a) unavailability of device to use the connected brush and (b) issues around safety and sustainability) raise important questions on how to best adapt and deliver such interventions. Mobile phone use is on the rise globally across all demographic and age groups, in the UK, 25% of children aged 5–7-years old now own a smartphone [33]. Despite the availability of smartphones for younger children and the barrier that many parents identified in their children not having their own mobile phone device to access and use the app, a key question of whether young children should own a smartphone in the first place remains. Children’s mental health, behaviour and sleep can suffer detrimental effects through prolonged mobile phone use and screen time exposure [34, 35] Restricting the introduction of a mobile phone device and generally, limiting the screen time exposure children receive each day is of paramount important for their overall wellbeing [33]. Therefore, it is crucial to consider how digital oral health interventions with connected devices can still be of use for children without adding unnecessary screen exposure to their daily lives nor creating pressure in families for children to own a smartphone.

### Limitations & strengths

This study had several limitations that need to be addressed in future work. Firstly, there was a loss of participants between consent and baseline measurements that may have led to some degree of non-response and desirability biases. This can partly be explained due to issues with participants’ emails with around 50 participants’ contact details not properly recorded in their self-completed, paper-consent forms to allow for emails with survey links to be sent to them. Moreover, the inclusion of electronic data collection methods could have led to reduced questionnaire completion rates compared to a combined approach using paper-based and electronic data collection. The requirement for households to have access to the internet and a smartphone could have unintentionally excluded some participants from the study however, internet connectivity and owning a smartphone were essential components of the study to allow for the use of the connected brush and app. Information provided to parents only in English could have led to exclusion for those parents whose English is not their first language. Not providing toothpaste to all students alongside the connected brush could have hindered some children’s ability to brush their teeth effectively if they lacked access to fluoride toothpaste. Compensation in the form of a shopping voucher could have inadvertently introduce a level of selection bias for participants. The pragmatic scope of the study could have added additional limitations to our overall results. One of the interviews was in Urdu and translated in English by one of the researchers who is a native Urdu speaker. That interview was not then back translated as per WHO guidelines presenting a potential, minor limitation.

Despite some limitations, this study had some key strengths. For example, working closely with the OHIT of MCC allowed us to identify and approach schools with the highest need for this intervention. The demographic composition of our sample was very diverse reflecting the areas where our study took place. Also, since all children in our targeted schools/classes received the brush regardless of consent, the intervention had a universal reach and offered potential benefits for everyone despite not being part of the study. Time commitment and overall burden to participants was low as reflected by only a single participant actively dropping out from the study. Overall consent rates remained in line, and even higher than contemporary work in children’s oral health reflecting another major strength of the study design.

### Future directions

Based on our primary feasibility outcomes, these were achieved at the end of the study. There were important issues that need to be addressed before moving forward with a definitive trial especially around consent and response rates. To achieve increased consent rates, a more involved recruitment approach should be considered where all schools receive additional, practical support in informing students and parents about the study. Also, additional checks should be in place to avoid issues with participants’ contact details and make sure that all participants can be reached in order for them to complete their follow-up surveys. Harnessing community services and involving local communities in general should remain a priority for any future studies. Providing additional support to families, for example, providing them with toothpaste or offering support for those who do not have a smartphone or internet connectivity should be considered. Finally, consideration should be given to language barriers throughout the study, not just for informed consent and creating a more inclusive intervention that is tailored to different community needs.

## Conclusions

This feasibility study was able to approach and recruit participants from underserved areas in Greater Manchester through primary schools with the active support of the local council and community oral health improvement teams. Despite losing participants between consent and baseline measurements, this loss could be easily mitigated through the use of non-electronic data collection techniques to complement electronic data collection in the future. Very positive feedback from parents and teachers highlights the potential of this intervention. Future studies should consider how to quantify changes in children’s oral health, address loss of questionnaire completion and harness the opportunities this study provided.

## Supplementary Information

Supplementary Material 1.

Supplementary Material 2.

## Data Availability

The datasets used and/or analysed during the current study are available from the corresponding author on reasonable request.

## Abbreviations

GM: Greater Manchester
GA: General Anaesthetic
MCC: Manchester City Council
OHIT: Oral Health Improvement Team
