# Supplementary figures and images for: Exploring the potential of a school brushing program using a connected brush in underserved areas: a feasibility cluster randomised trial
Source: BMC Oral Health. 2025 Feb 11;25:223. doi: 10.1186/s12903-025-05573-7 (PMC11817162; doi:10.1186/s12903-025-05573-7)

Appendix 2. Logic Model Leapfrog


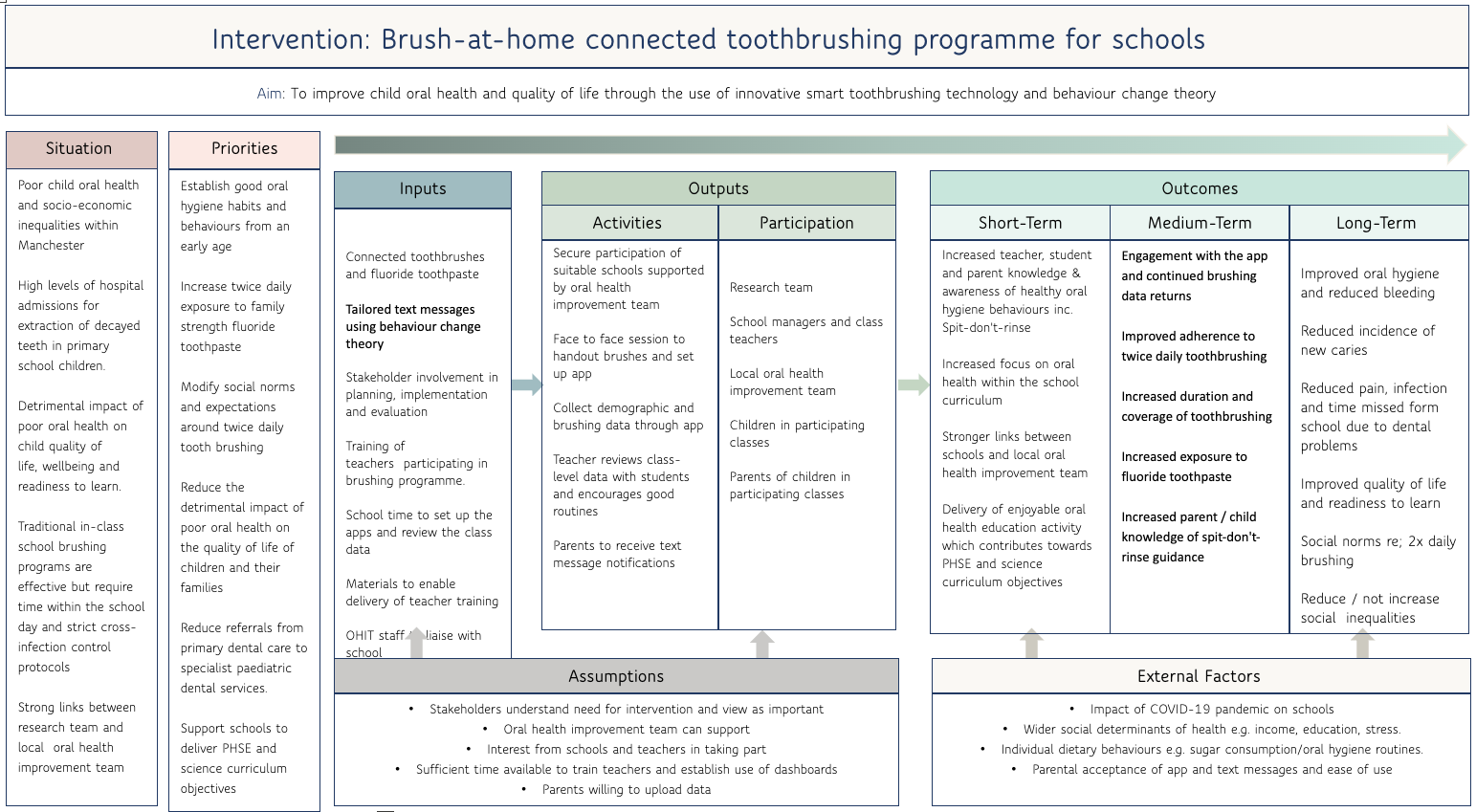

Supplement: Supplementary file 2 — Supplementary Material 2. [file 12903_2025_5573_MOESM2_ESM.docx]
